# Immunological response to highly active antiretroviral therapy following treatment for prevention of mother to child transmission of HIV-1: a study in Côte d'Ivoire

**DOI:** 10.1186/1758-2652-13-28

**Published:** 2010-08-02

**Authors:** Didier K Ekouevi, Patrick A Coffie, Marie-Laure Chaix, Besigin Tonwe-Gold, Clarisse Amani-Bosse, Valériane Leroy, Elaine J Abrams, François Dabis

**Affiliations:** 1 Centre de recherche, Inserm U 897, Bordeaux France; 2 Institut de Santé Publique, Epidémiologie et Développement, Université Victor Segalen Bordeaux 2, Bordeaux, France; 3 ANRS DITRAME PLUS Project, Programme PAC-CI, Abidjan, Côte d'Ivoire; 4 Université René Descartes, EA 3620, Assistance Publique - Hôpitaux de Paris, Laboratoire de Virologie, Hôpital Necker Enfants Malades, Paris, France; 5 MTCT-Plus initiative, ACONDA, Abidjan, Côte d'Ivoire; 6 MTCT-Plus Initiative, International Center for AIDS Care and Treatment Programs, Mailman School of Public Health, Columbia University, New York, NY, USA

## Abstract

**Background:**

Information is currently limited on the long-term follow up of HIV-1 infected women who are on highly active antiretroviral therapy (HAART) that contains nevirapine and lamivudine and who were previously exposed to antiretroviral drugs for the prevention of mother to child transmission (PMTCT) of HIV.

**Methods:**

We studied the 36-month immunological response to HAART in HIV-1 infected women in Côte d'Ivoire. The women were previously exposed to antiretroviral drug regimens for PMTCT, including single-dose nevirapine and/or short-course zidovudine with or without lamivudine. All HAART regimens included a non-nucleoside reverse transcriptase inhibitor.

**Results:**

At 36 months: the median absolute increase in CD4+ T cell count was +359 cells/mm^3 ^(IQR: 210-466) in 200 women who had undergone 36-month follow-up visits; +359 cells/mm^3 ^(IQR: 222-491) in 88 women not exposed to PMTCT antiretrovirals; and +363 cells/mm^3 ^(IQR: 200-464) in 112 women exposed to at least one antiretroviral PMTCT regimen. Overall, 49 (19.8%) of the 247 women who initiated HAART met the immunological failure criteria at least once during follow up. The overall probability of immunological failure was 0.08 (95% CI: 0.12-0.15) at 12 months, and 0.21 (95% CI: 0.16-0.27) at 36 months. No difference was observed according to the presence or absence of resistance mutations to nevirapine or lamivudine in women tested at four weeks postpartum. In addition, at 36 months, 23% of women were lost to follow up, dead or had stopped their treatment.

**Conclusions:**

A non-nucleoside reverse transcriptase inhibitor-based antiretroviral regimen, initiated a year or more after PMTCT exposure and that includes nevirapine, remains a good option for at least the first 36 months of treatment.

## Background

Information is currently limited on the long-term follow-up of HIV-1 infected women who are on highly active antiretroviral therapy (HAART) containing nevirapine (NVP) and lamivudine (3TC) and who were previously exposed to antiretroviral (ARV) drugs for the prevention of mother to child transmission (PMTCT) of HIV [1-4].

A 12-month study showed a good immunological response in women previously exposed to ARV drugs for PMTCT [2]. However, in this study we found a higher risk of virological failure in women who had 3TC-acquired resistance mutation four weeks postpartum [2]. We now report the immunological response to HAART at 36 months in women previously exposed to short-course ARV prophylaxis and study factors associated with immunological failure or with immunological failure and death according to the history of PMTCT exposure.

## Methods

A prospective cohort study was conducted in Abidjan, Côte d'Ivoire, between August 2003 and June 2009 among all HIV-infected women who initiated HAART in the MTCT-Plus initiative. The study population and study design have previously been described [2].

Very briefly, our population consisted of: (1) women never exposed to any treatment for PMTCT; (2) women exposed to single-dose NVP (sdNVP) and zidovudine (ZDV) for PMTCT; and (3) women exposed to short-course zidovudine (scZDV) and 3TC for PMTCT. The primary variable of interest was the presence of viral resistance mutation to NVP or 3TC measured at Week 4 postpartum.

Two outcomes were considered after 36 months on HAART: (1) immunological failure, defined as a 50% fall from absolute CD4+ T cell count peak level [5]; and (2) a combined criteria, defined as either immunological failure or the occurrence of death during the first 36 months of follow up. The virological analyses were done retrospectively, and results were not available for clinical use. Decisions to switch antiretroviral regimens were thus made by local clinicians based on routinely collected immunological and clinical data.

Other study variables measured at time of HAART initiation were included in the analysis: age, WHO clinical stage, body mass index, hemoglobinemia at HAART initiation, and self-reported adherence (seven-day self-report at six, 12, 18, 24, 30 and 36 months) [5]. Cox regression was used to identify factors associated in univariable analysis (p < 0.20) with immunological failure or the combined criteria. We censored the follow up of each patient at the date of last visit, or death, or date of switch to a protease inhibitor (PI) to evaluate the response to non-nucleoside reverse transcriptase inhibitor (NNRTI)-based treatment.

## Results

From August 2003 to September 2005, 247 women initiated 3TC-containing HAART with either NVP or efavirenz (EFV). At HAART initiation, their median age was 28 years (interquartile range: 25-32) and their median CD4+ count was 188 cells/mm^3 ^(IQR: 126-264). Overall, 28 women (11.3%) were classified at WHO clinical Stage 1; 110 (44.5%) at Stage 2; 96 (38.9%) at Stage 3; and 13 (5.3%) at Stage 4. A total of 109 women (44.1%) had never been exposed to a PMTCT ARV regimen during a previous pregnancy, and 138 (55.9%) had previously received a PMTCT regimen: 50 had received scZDV + sdNVP and two sdNVP only; 81 had received scZDV+sc3TC+sdNVP and five scZDV+3TC only. Among 73 of the 86 3TC-exposed women tested for resistance mutations at Week 4 postpartum, 11 (15.1%) had detectable 3TC resistance mutations. Among 111 of the 133 sdNVP-exposed women tested at Week 4 postpartum, 19 (17.1%) had detectable NVP resistance mutations.

The first-line HAART regimen was ZDV+3TC+NVP in 234 (95.1%) women, and stavudine (d4T)+3TC+NVP in seven (2.9%) women; five women started HAART with ZDV+3TC+EFV and one began with d4T+3TC+EFV. The median time between exposure to sdNVP and initiation of HAART was 21 months (IQR: 13-26). During the 36 months of follow up, 30 women (12.1%) were lost to follow up, 10 (4.0%) stopped treatment at their own request, and 17 (6.9%) died. Furthermore, 19 out of 247 (7.7%) HIV-infected women switched to PI-based HAART. The reasons for switching were immunological failure in four patients and side effects related to NVP or EFV in 15 cases. There was no association between switching to PI and PMTCT-acquired resistance to NVP (p = 0.43) or to 3TC (p = 0.93).

**Figure 1:**
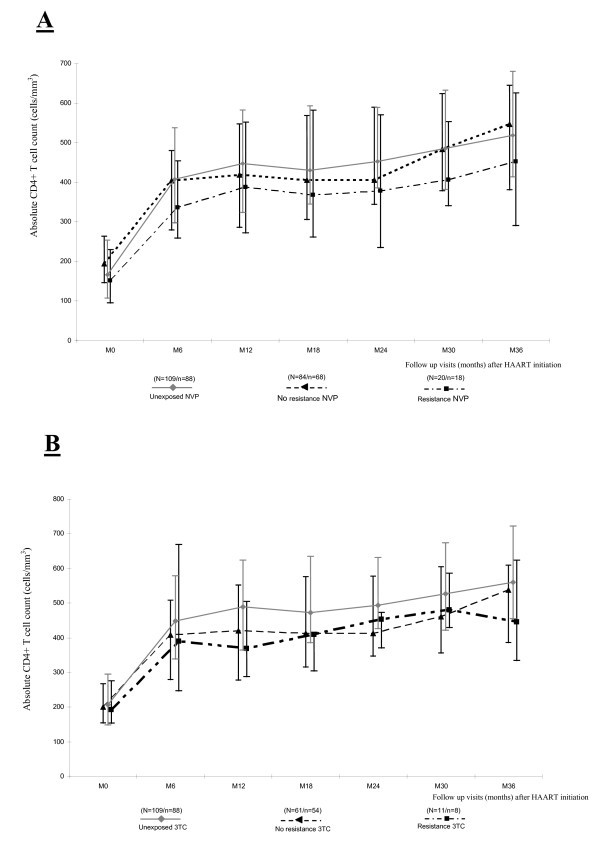
**Immunological response in HIV-infected women**. A. Immunological response in HIV-infected women exposed to nevirapine or acquired PMTCT resistance to nevirapine. B. Immunological response in HIV-infected women exposed to lamivudine or acquired PMTCT resistance to lamivudine. NVP = nevirapine. 3TC = lamivudine. N = number of patients with CD4 count available at baseline (M0). n = number of patients with CD4 count available at month 36 (M36).

At 36 months, the median absolute increase in CD4+ count was +359 cells/mm^3 ^(IQR: 210-466) in 200 women who had undergone 36-month follow-up visits. The increase was +359 cells/mm^3 ^(IQR: 222-491) in 88 women not exposed to PMTCT ARV, and +363 cells/mm^3 ^(IQR: 200-464) in 112 women exposed to at least one ARV PMTCT regimen. The median absolute increase in CD4 count was +366 cells/mm^3 ^(IQR: 174-461) in the 18 women with NVP resistance at Week 4 postpartum, and > 230 cells/mm3 (IQR: 130-403) in the eight women with 3TC resistance mutations detected at Week 4 postpartum (Figures 1A and 1B). When analyses of immunological response were restricted to the 184 women who did not switch to a PI during the follow up, the median absolute increase in CD4+ count was 361 cells/mm^3 ^(IQR: 220-466) at 36 months.

Overall, 49 (19.8%), (95% CI 15.0-25.3%) of the 247 women who initiated HAART met the immunological failure criteria at least once during follow up. Regarding immunologic failure, no statistical difference was found between the women unexposed to sdNVP, those exposed to NVP without resistance mutations, and those with NVP resistance mutations at Week 4 postpartum (14.7% vs. 19.8% vs. 25.0%, respectively, p = 0.50). The same conclusion was drawn for 3TC exposure (14.7% in women unexposed to 3TC vs. 14.8% in women exposed without 3TC-resistance mutation vs. 18.2% in women exposed with 3TC-resistance mutations, p = 0.71).

The overall probability of immunological failure was 0.08 (95% CI: 0.12-0.15) at 12 months, 0.14 (95% CI: 0.10-0.19) at 24 months and 0.21 (95% CI: 0.16-0.27) at 36 months. At 36 months, the probability of immunological failure was 0.25 (95% CI: 0.18-0.28) in women who selected a 3TC-resistant virus after PMTCT and 0.21 (95% CI: 0.14-0.31) in women who selected a NVP-resistant virus.

For the second outcome (death or immunological failure), the probability at 36 months was 0.26 (95% CI: 0.20-0.31) in the overall population. It was 0.32 (95% CI: 0.16-0.58) in women who had NVP resistance mutations at Week 4, and 0.30 (95% CI: 0.11-0.68) in women with 3TC resistance mutation at Week 4.

In multivariate analysis (n = 247), the only factor associated with immunological failure was poor self-reported adherence (adjusted Hazard Ratio, aHR, 2.61; 95% CI 1.43-4.74, p = 0.002), controlling for resistance mutations and exposure to NVP or 3TC, CD4 count, maternal age, WHO clinical stage, and hemoglobinemia at HAART initiation. Self-reported adherence was also associated with the combined criteria (aHR 4.14; 95% CI 2.39-7.19, p < 0.001).

## Discussion

During the 36 months of follow up, we did not find any differences for immunological failure related to the presence or absence of NVP- or 3TC-resistance mutations at Week 4 postpartum. Our findings are consistent with those reported by others for shorter periods of follow up [1-4,6], and support the recommendation to use NNRTI-based regimens in women previously exposed to NVP when the delay between the exposure to NVP and HAART initiation is longer than 12 months [6]. The long delay between PMTCT exposure and HAART initiation in our study likely resulted in the fading of detectable resistance mutations acquired with PMTCT ARV exposure [7,8].

We also reported that 7.7% of the women switched from NNRTI-based to PI-based HAART during follow up. Among the 19 women who switched to PI-based HAART, only four switches were related to treatment failure; the other 15 were done to manage NNRTI-related drug toxicity. While 49 (19.8%) women met immunologic criteria for failure at least once during follow up, very few were changed to second-line therapy. Similar findings have been reported in the *Médecins Sans Frontières *multi-country cohort, with an incidence of switch of 4.8 per 1000 person -years [9]. Reasons that patients did not switch were not recorded, but we hypothesize that this is a common practice in settings with limited availability of ARV drugs, and where physicians often choose to reinforce adherence and postpone regimen changes in clinically stable patients.

Two limitations are noted. First, there is a lack of viral load data, which is not routinely monitored and recorded in our study area. Such data is important to fully understand the dynamics and rate of treatment failure in our population. Switching treatments without using viral load data for making these decisions is indeed of utmost concern [10,11]. Second, the limited sample size implies limited statistical power to detect any immunologic difference between the groups of interest variable studied. However, our results were consistent with previous reports [3,4,6].

In addition, 23% of women were lost to follow up, dead or had stopped treatment at 36 months, and were no longer on treatment despite the establishment of a well-funded programme with excellent resources [12]. However, very few data are available on long-term follow up of ART treatment in low income-countries to make any comparison between studies. In sub-Saharan Africa, 38% of patients were lost to care and therefore no longer on treatment after two years of follow up [13].

## Conclusions

In conclusion, an NNRTI-based antiretroviral regimen, which includes NVP, initiated at least one year after PMTCT exposure remains a good option for at least the first 36 months of treatment. Larger studies, preferably with virological and genotypic test data, are needed to confirm our findings and to better decide when to switch HAART regimens.

## Competing interests

The authors declare that they have no competing interests.

## Authors' contributions

DKE, PAC and FD designed the study. PAC and CAB collected the data. DKE and PC analyzed the data. DKE and PAC interpreted the data. All authors contributed to the writing of the manuscript, and all authors approved the manuscript for publication.
